# Identification of a virulence-related surface protein XF in piscine *Streptococcus agalactiae* by pre-absorbed immunoproteomics

**DOI:** 10.1186/s12917-014-0259-7

**Published:** 2014-10-26

**Authors:** Guangjin Liu, Wei Zhang, Yongjie Liu, Huochun Yao, Chengping Lu, Pao Xu

**Affiliations:** Key Laboratory of Animal Bacteriology, Ministry of Agriculture, Nanjing Agricultural University, Weigang No.1, Nanjing, 210095 Jiangsu China; Key Laboratory of Freshwater Fisheries and Germplasm Resources Utilization, Ministry of Agriculture, Freshwater Fisheries Research Center, Chinese Academy of Fishery Sciences, Wuxi, 214081 China

**Keywords:** *Streptococcus agalactiae*, Piscine, Pre-absorbed immunoproteomic method (PAIM), Serine-rich repeat protein (Srr), Zebrafish

## Abstract

**Background:**

Since 2009, large-scale *Streptococcus agalactiae* infections have broken out in cultured tilapia farms in China, resulting in considerable economic losses. Screening of the surface proteins is required to identify virulence factors or protective antigens involved in piscine *S.agalactiae* infections in tilapia. Pre-absorbed immunoproteomics method (PAIM) is a useful method previously established in our laboratory for identifying bacterial surface proteins.

**Results:**

A serine-rich repeat protein family 1 (Srr-1), designated XF, was identified by PAIM in piscine *S. agalactiae* isolate GD201008-001. To investigate the role of XF in the pathogenesis of piscine *S. agalactiae*, an isogenic *xf* mutant strain (Δ*xf*) and a complemented strain (CΔ*xf*) were successfully constructed. The Δ*xf* mutant and CΔ*xf* showed no significant differences in growth characteristics and adherence to HEp-2 cells compared with the wild-type strain. However the 50% lethal dose of Δ*xf* was increased (4-fold) compared with that of the parental strain in a zebrafish infection model.

**Conclusions:**

The findings demonstrated that XF is a virulence-related, highly immunoreactive surface protein and is involved in the pathogenicity of *S. agalactiae* infections in fish.

**Electronic supplementary material:**

The online version of this article (doi:10.1186/s12917-014-0259-7) contains supplementary material, which is available to authorized users.

## Background

*Streptococcus agalactiae*, also referred to as Group B Streptococcus (GBS), is a Gram-positive, β-hemolytic, chain-forming coccus. It has been recognized as one of the major causes of pneumonia and meningitis in neonates [1], mastitis in cows [2], and meningoencephalitis in fish [3]. Since 2009, serious piscine GBS infections have occurred among tilapia farms in China, causing high mortality and resulting in considerable economic losses [4]. The isolate GD201008-001, a highly virulent strain isolated from a moribund cultured tilapia in China, was identified and sequenced in our laboratory in 2012 [5]. According to our comparative genomics analysis [6], isolates from cultured tilapia in China have a very close genomic relationship with the human strain A909. In fact, the genomic content of these isolates cannot be distinguished from that of A909. *S. agalactiae* A909 was originally isolated from the cerebrospinal fluid of a neonate with meningitis in the USA [7]; therefore, *S. agalactiae* infections among fish in China may represent a zoonotic hazard although further investigations are required to confirm this hypothesis.

As a consequence of the potential economic and ecological impacts on public health, many studies have been conducted on *S. agalactiae* with the aim of identifying virulence factors [8], diagnostic markers [9,10] and treatments [11]. Surface proteins of pathogenic bacteria are known to mediate the key step in the bacteria-host interaction during infection and may represent protective antigens and virulence markers. Thus, identification of *S. agalactiae* surface proteins associated with bacterial infection and a greater understanding of their roles in pathogenicity are required. The pre-absorbed immunoproteomics method (PAIM) was successfully established as a useful tool for identifying bacterial surface proteins in our previous study [12,13] and has been applied in *Escherichia coli* [14] and *Campylobacter jejuni* [15]. This technique involves the separation of proteins by two-dimensional electrophoresis (2-DE) and Western blotting with untreated (hyperimmune rabbit serum) and “pre-absorbed” serum, which is produced from untreated serum using a modified cross-absorption process to remove bacterial surface antigen-specific antibodies. Protein spots that appear in the blot probed with untreated sera, but that are absent in the blot probed with pre-absorbed sera, are assumed to be surface proteins.

In this study, we performed PAIM analysis of the piscine *S. agalactiae* strain GD201008-001 and identified a serine-rich repeat protein (Srr-1), designated XF, among the potential surface proteins. Previous research has indicated that Srr-1 is an important surface protein for adherence and virulence in human *S. agalactiae* strains [16,17]. However, the role of Srr-1 in the pathogenicity of piscine *S. agalactiae* is unknown. To investigate this issue, a mutant strain (Δ*xf*) and a complemented strain (CΔ*xf*) were constructed using *streptococcus-E. coli* shuttle vectors pSET4s [18] and pSET2 [19]. Their growth characteristics, adherence to HEp-2 cell and virulence were evaluated in a zebrafish infection model.

## Results and discussion

### Pre-absorbed immunoproteomics on S. agalactiae

**Figure 1:**
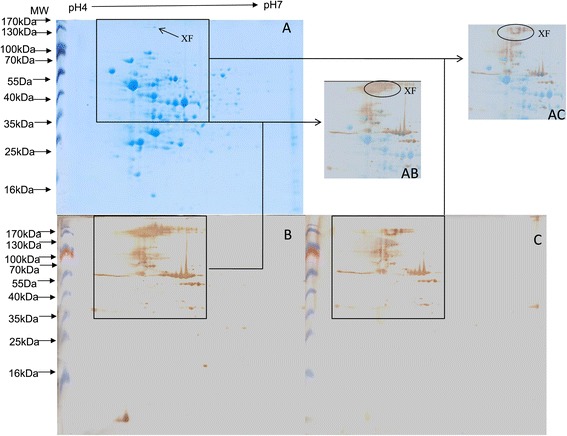
**Pre-absorbed immunoproteomics on picsine** ***S. agalactiae*** **GD201008-001. (A)** GD201008-001 total cell proteins (pH 4–7, 13 cm), stained with Coomassie brilliant blue G-250. **(B)** 2-DE blot of GD201008-001 proteins probed with untreated antiserum. **(C)** 2-DE blot of GD201008-001 proteins probed with “pre-absorbed” antiserum. **(AB)** Merge of layers A and B. In Photoshop CS, the duplicated gel layer A was used as the background layer and the western blotting layer B as the surface layer in 50% transparency. **(AC)** Merge of layers A and C. In Photoshop CS, the duplicated gel layer A was used as the background layer and the western blotting layer C as the surface layer in 50% transparency.

Untreated and pre-absorbed antisera were used to probe 2-DE blots of *S. agalactiae* GD201008-001 cell lysates. The immunoreactive spots observed in immunoblots (Figure 1B and C) were matched with the corresponding protein spots observed in the stained 2-DE gel (Figure 1A) by comparison using the layer function of Photoshop CS. In order to minimize the proteomics trial error, the experiments were repeated at least in triplicate, and only the highly reproducible spots were selected. One spot, XF, that invariably appeared in the blot probed with untreated serum (Figure 1AB), but was less distinct in the blot treated with pre-absorbed serum (Figure 1AC) in the triplicate repeat tests, was excised from preparative 2-DE gels, subjected to tryptic digestion, and then analyzed by matrix-assisted laser desorption ionization-time of flight mass spectrometry (MALDI-TOF MS) and the PSORT program. Other spots which were not detected using the pre-absorbed serum in repeat experiments were also excised and analyzed by MALDI-TOF MS; however, these proteins were predicted to be cytoplasmic by the PSORT program (data not shown).

Because of the much greater sensitivity of western blotting, the XF spot was more extensive in these blots than in the paired Coomassie brilliant blue G-250 stained gels. Consequently, low abundance proteins with strong immunoreactivity, such as XF, are strongly stained in western blotting analyses. In addition, the serine-rich repeat proteins of *S. agalactiae* have been reported to be high MW glycoproteins (300–400 kDa) due to glycosylation [17,20], although their genes encode just 654–1,326 amino acids. In the present study, the apparent molecular weight of XF was in the 150 kDa range. It can be speculated that the large disparity between the published reports and our result is due to differences in the degree of glycosylation of serine-rich repeat proteins. Furthermore, one of the limitations of 2-DE is the inability to analyze very large (MW >200 kDa) or very small (MW <10 kDa) proteins [21]. Consequently, fully glycosylated serine-rich repeat proteins (MW >300 kDa) will not be detected in 2-DE.

### The Srr-1 protein from piscine and human S. agalatiae strains

**Figure 2:**
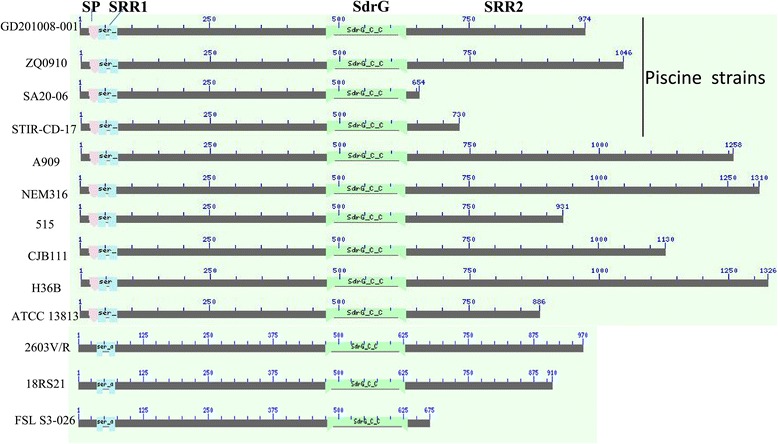
**Alignment of the Srr-1 proteins from piscine, bovine and human** ***S. agalatiae*** **strains.** Alignment was performed using conserved domain architecture retrieval tool on NCBI. SP: signal peptide; SRR1: serine-rich repeat domain 1; SdrG: fibrinogen-binding domain; SRR2: serine-rich repeat domain 2.

Spot XF was identified as a cell wall surface anchor family protein (gi|406709853) by MALDI-TOF MS (Additional file 1), also known as a serine-rich repeat protein (Srr-1) [22]. It has been confirmed that Srr-1 is localized on the surface of streptococcal cells and binds to human fibrinogen and keratin [23]. Previous immunoproteomics studies have confirmed that Srr-1 is a highly immunoreactive protein that is reactive with both *S. agalactiae* convalescent guinea pig sera and infected tilapia antisera [24]. The conserved domain architecture retrieval tool of NCBI (Figure 2) showed that the Srr-1 protein has an orthologous counterpart among GBS strains: a long and specialized signal sequence, two extensive serine-rich repeat regions (SRR1, 2) that undergo glycosylation, a fibrinogen-binding domain (SdrG), which is necessary for binding to human fibrinogen and keratin 4 [23], and a typical LPXTG cell wall anchoring motif. The Srr-1 proteins range in size from 654 aa to 1,326 aa and the C-terminal SAS (T/M) repeat region (SRR2) is responsible for the observed size variation, which has also been reported previously by Samen et al. [16]. Blastp analysis showed that the similarity of the amino acid sequence of Srr-1 proteins from 1 aa to 643 aa (containing a SdrG) is greater than 90%, and 100% identical among piscine strains GD201008-001 and ZQ0910 and human strains A909 and CJB111. Protein XF contains 974 aa, with a SdrG region between 472–629 aa and a SRR2 region from 698–974 aa. In piscine *S. agalactiae* SA20-06, the Srr-1 protein is only 654 aa and devoid of SRR2 due to a single base (A) deletion at the position 1,917 bp in its ORF leading to early termination. In the study by Takahashi et al. [25], the predicted secondary structures of Srr proteins from *S. agalactiae* are similar to those of Srr proteins from other streptococcal species. Using the SWISS-MODEL tool, we found that the predicted secondary structures of Srr-1 proteins among *S. agalactiae* were similar, even when compared with strain SA20-06.

### Construction of the mutant and complementation strains

**Figure 3:**
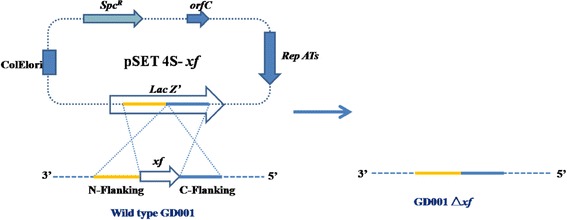
**Schematic representation of construction of** ***xf mutant in S. agalactiae*** **GD201008-001 by allelic exchange.** Representation of the chromosomal structures before (left) and after (right).

To further investigate the function of protein XF in the pathogenesis of piscine *S. agalactiae*, the plasmids pSET4s-XF and pSET2-CΔ*xf* were constructed as *xf* gene deletion (Figure 3) and complementation mutants, respectively.

**Figure 4:**
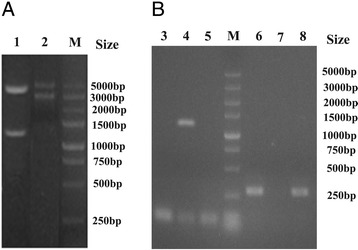
**Restriction enzyme digestion identification of recombinant plasmids (A) and PCR confirmation of knockout mutant strain Δ** ***xf*** **(B).** Lane 1: pSET4S-*xf* plasmid enzyme digestion; Lane 2: pSET2-CΔ*xf* plasmid enzyme digestion; Lane 3: primers XF A/D, GD201008-001; Lane 4: primers XF A/D, ΔXF; Lane5: primers XF A/D, CΔ*xf*; Lane 6: primers XF 1/2, GD201008-001; Lane 7: primers XF1/2, Δ*xf*; Lane 8: primers XF 1/2, CΔ*xf*; M: Marker DL5000.

**Figure 5:**
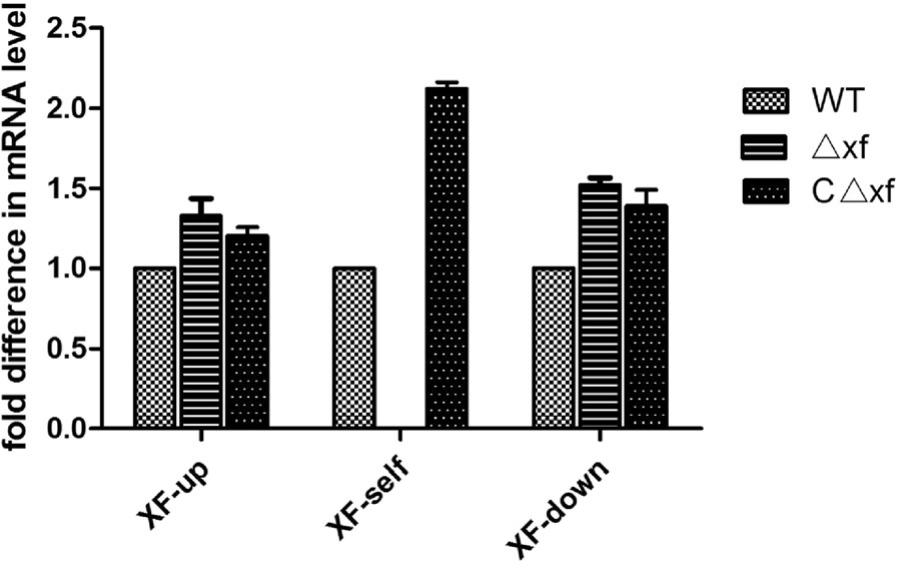
**Transcription level of XF upstream, downstream and XF self open-reading frames between the wild-type and its derivatives in** ***vitro*** **.** The value of *xf* associated genes in the wild-type is set as 1.0. The relative change in the gene transcription ratios of the selected genes was normalized to the transcription of a single housekeeping gene (GAPDH gene), and calculated as by the 2^–ΔΔ CT^ method. Data are representative of three independent experiments carried out in triplicate. Error bars indicate standard deviations.

The pSET4s-XF and pSET2-CΔ*xf* plasmids were verified to be correct by diagnostic restriction enzyme digestion with *Sal*I/*Sma*I and *BamH*I/*Pst*I, respectively (Figure 4A). The mutant strains Δ*xf* and CΔ*xf* were confirmed by PCR (Figure 4B) and real-time PCR (Figure 5). The primers XF A and D amplified a 1,377 bp fragment of the Δ*xf*, while no product was generated from the parental and complementation templates. The XF1/XF2 primers amplified a 289 bp product from the parental GD201008-001 and CΔ*xf* templates while no product was obtained from the Δ*xf* template. All the PCR products obtained had the expected fragment sizes (Figure 4B) and in correct sequence.

To confirm that the mutant has no effect on the transcription of bilateral genes, qRT-PCR assays was performed to detect the expression of *xf* gene, *xf* upstream and downstream ORF genes in the parental, mutant and complementation strains. Data for the three strains were normalized to the expression of the GAPDH gene using the 2^−ΔΔCT^method. The transcription levels of *xf* gene in the Δ*xf* were below the level of detection for this assay and there were no significant differences in *xf* upstream and downstream gene transcription levels between Δ*xf* and CΔ*xf* (*P* >0.05; Figure 5). These results indicated the successful construction of an isogenic knockout mutant of *xf* and complementation of Δ*xf,* with no effects on the transcription of bilateral genes. The transcription level of *xf* in the CΔ*xf* strain replicated from plasmid pET-2 was higher than that replicated from the genome of the wild-type strain (Figure 5) due to the increased plasmid copy number compared to the genome replication number, with each new cell acquiring at least one copy of the plasmid as the cell divides. Moreover, the plasmid pET-2 was constructed from pSSU1, a native plasmid isolated from *S. suis* DAT1 [19] and belonging to the pMV158 plasmid family [26]. Lorenzo-Diaz et al. showed that the number of copies of the pMV158 vector was lower in enterococci (around 17 copies per genome equivalent) than that in pneumococci (around 30 copies) [27].

### Growth characteristics of the mutant and complementation strains

**Figure 6:**
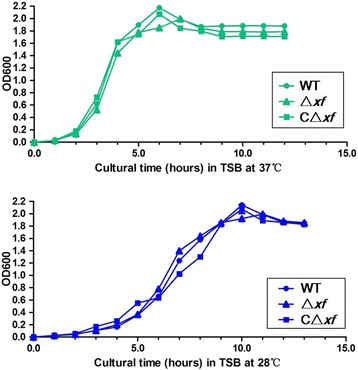
**Growth curve of** ***S. agalactiae*** **GD201008-001 and its derivatives at 37°C and 28°C.**

Before studying the effect of XF inactivation on the pathogenesis of *S. agalctiae* in vivo, it is important to characterize the growth characteristics of the parental, mutant and complementation strains in vitro. The OD_600_ of cultures of the GD201008-001 and Δ*xf* strains in TSB broth and CΔ*xf* in TSB broth containing 100 μg/mL spectinomycin at 37°C were determined. In addition, the growth curves of these strains at 28°C were also determined for evaluation of the LD_50_ values in a zebrafish infection model at 28°C. As shown in Figure 6, there were no significant differences in the growth curves of the wild-type, Δ*xf* and CΔ*xf* strains at the same temperature. The bacterial population reached a peak during the logarithmic growth phase after 6 h at 37°C and after 10 h at 28°C. Moreover, no differences in shape and chain formation were observed between the strains following Gram staining (data not shown).

### Contribution of XF to adhesion and invasion in vitro

**Figure 7:**
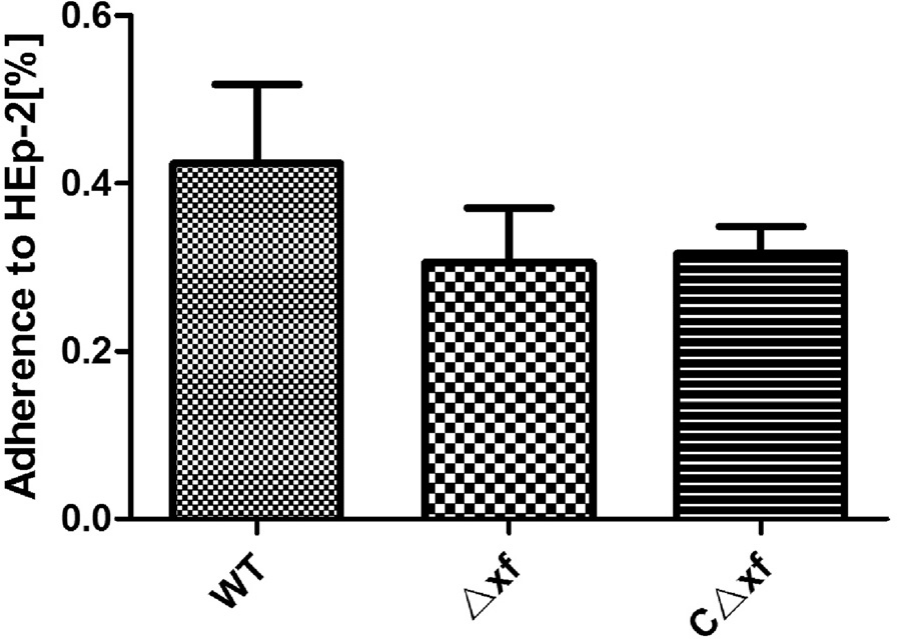
**Effects of** ***xf*** **on** ***S. agalactiae*** **adherence to HEp-2 cells (MOI: 10).** Data are representative of three independent experiments carried out in triplicate. Error bars indicate standard deviations.

To investigate the importance of XF for bacterial adherence to, and invasion of host cells, the human epithelial cell line HEp-2 was infected in vitro with the parental, mutant and complementation strains under the same conditions in triplicate repeat tests. As shown in Figure 7, the adherence of the GD201008-001 wild-type strain to HEp-2 cells was only 0.4% with a multiplicity of infection (MOI) of 10 and there were no significant differences in the adherence to HEp-2 cells among the GD201008-001, Δ*xf* and CΔ*xf* strains (*P* >0.05). In the invasion assays, the parental strain GD201008-001 displayed poor invasion of HEp-2 cells (<0.0005%) and there were no significant differences in corresponding activity exhibited by the Δ*xf* and CΔ*xf* strains (data not shown). These results indicate that piscine *S. agalactiae* GD201008-001 has weak capacity for adherence to and invasion of HEp-2 cells. Thus, XF has almost no effect on bacterial adherence and invasion to HEp-2 cells under the chosen experimental conditions.

The study by Samen et al. showed that Srr-1 could not mediate bacterial invasion into HEp-2 cells, but promoted adherence of the *S. agalactiae* human strain 6313 to HEp-2 cells [16]. Compared with our results, Srr-1 showed a different effect on adherence to Hep-2 cells between human and piscine strains. Since piscine *S. agalactiae* GD201008-001 showed only weak adhesion capability to HEp-2 cells, this cell line may not the best choice for investigation of the cell adhesion capacity of piscine *S. agalactiae*. The contribution of XF to adhesion and invasion in vitro was not clearly reflected under these experimental conditions; therefore, development of an alternative host cell, such as a tilapia epithelial cell line, is required for more accurate evaluation of the adhesion capability of piscine bacteria in vitro. It can be speculated that the poor capacity of the piscine strain for adherence to and invasion of HEp-2 cells is the result of the loss of some genes nonessential genes involved in adherence to human cells during adaptation to the fish host. For example, there are no *lmb* or *scpB* genes derived from the genomes of piscine *S. agalactiae* strains in the NCBI nr database to date [28]. These two genes encode two important adhesins in human strains: laminin-binding protein and C5a peptidase, respectively [29,30].

### Virulence of wild-type and Δxf strains in a zebrafish infection model

**Figure 8:**
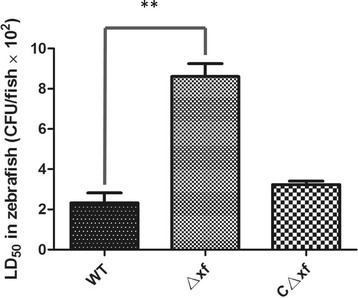
**LD**_**50**_ **of wild-type, mutant and complementation strains in the zebrafish infection model.**

The zebrafish infection model was used to estimate the effect of the XF mutation on *S. agalactiae* virulence. This model has already been established for multiple streptococcal infections, including *Streptococcus pyogenes* [31], *Streptococcus pneumonia* [31], *Streptococcus suis* [32] and *Streptococcus agalactiae* [33]. Zebrafish have many advantages for this purpose, such as the existence of both innate and adaptive immune systems, short generation time, ease of breeding large numbers in the laboratory and suitability for large-scale genetic screens. Zebrafish were injected intraperitoneally (i.p.) with either the parental GD201008-001, Δ*xf* or CΔ*xf* strains at various doses in triplicate repeat tests. The mortality of zebrafish was observed within 7 days post-challenge. As shown in Figure 8, the average LD_50_ values determined in triplicate assays were 2.3 × 10^2^ CFU/zebrafish for the wild-type strain, 8.6 × 10^2^ CFU/zebrafish for Δ*xf*, and 3.3 × 10^2^ CFU/zebrafish for CΔ*xf*. The 50% lethal dose of Δ*xf* was increased (4-fold) compared with that of the parental strain while that of the complementation strain CΔ*xf* was restored to 0.4-fold higher than that of the mutant strain Δ*xf*. These results indicate a significant reduction in the virulence of *S. agalactiae* in the Δ*xf* mutant strain (*P* <0.05).

## Conclusions

In this study, a serine-rich repeat protein family 1 (Srr-1), designated XF, was identified by PAIM in the piscine *S. agalactiae* isolate GD201008-001. The effect of the XF protein on the virulence of piscine *S. agalactiae* in zebrafish was significant despite its low adherence to the human epithelial cell line Hep-2. In conclusion, our study indicates the involvement of Srr-1 in the virulence of *S. agalactiae* in fish.

## Methods

### Bacterial strains, plasmids and growth conditions

*Streptococcus agalactiae* strain GD201008-001 was isolated from a moribund cultured tilapia with meningoencephalitis in the Guangdong Province of China in 2010 [5]. The strain was grown in trypticase soy broth (TSB, Oxoid) at 37°C. *Escherichia coli* DH5α was supplied by Tiangen (Beijing, China). The *streptococcus-E.coli* shuttle vectors pSET4s and pSET2 were donated by Dr. Takamatsu from the Laboratory of Molecular Bacteriology, National Institute of Animal Health, Ibaraki, Japan. *E. coli* strains were maintained in Luria–Bertani (LB) broth at 37°C. Antibiotics (Sigma) were added to culture media as required at the following concentrations: ampicillin, 50 μg/mL for *E. coli*; spectinomycin, 100 μg/mL for *S. agalactiae*, 50 μg/mL for *E. coli*. Bacteria were stored as frozen cultures at −20°C in either TSB or LB, containing 25% (v/v) glycerol. All the strains and plasmids used in this study were showed in Table 1.

**Table 1:** **Characteristics of bacterial strains, plasmids and primers used in this study**

**Strains, plasmids and primer**	**Characteristics**	**References**
GD201008-001	Virulent strain of piscine *S.agalactiae* isolated in China	Collected in our laboratory
Δ*xf*	Mutation in *xf* gene of GD201008-001	In this study
CΔ*xf*	Complemented strain of Δ*xf*; *Spc* ^*R*^	In this study
*E. coli* DH5a	Cloning host for maintaining the recombinant plasmids	Invitrogen
Plasmids		
pSET4s vector	Temperature-sensitive *Streptococcus*-*E. coli* shuttle vector pSET4s; 4506 bp; *Spc* ^*R*^	[18]
pSET2 vector	Streptococcu- *E. coli* shuttle vector pSET2; 5016 bp; *Spc* ^*R*^	[19]
Primers		
XF A	CGC*GTCGAC*GAAGTTGTAAAGCGTTTG	In this study
XF B	TTTGGACATGTTTCCTCC, amplifies the flank sequence upstream *xf* coding sequence (665 bp)	
XF C	GGAGGAAACATGTCCAAAAAAGACTAAACCTACTTT	
XF D	TCC*CCCGGG*CCGAACGATTATACAT; amplifies the flank sequence downstream *xf* coding sequence (712 bp)	
C XF F	AAA*CTGCAG*ACACAACTCCTTTCACTCATT	
C XF R	CGC*GGATCC*GGCTTTTTACAAACTTCTACA; amplifies the structural gene of the *xf* gene, including its own promoter (3407 bp)	
XF 1	AATGATTGCAAGCGATA	
XF 2	GTTAACAGAAGCGATTGA; amplifies the fragment inside the *xf* gene(289bp)	
RT-PCR Primers		
GAPDH-F	GATGACTACTATCCACGCATACAC	In this study
GAPDH-R	TGCAGCACCAGTTGAGTTAG	
XF up F	CCTGTGTTGGAGTGAAGATAGAG	
XF up R	CCAGCAGCCAAGAAAGTAGATA	
XF self F	CTTGCAGAACAAACGGAAGTG	
XF self R	TGAGGCTGACTCTGACATAGA	
XF down F	CTCGTTCTTCTGTCTATCGTCTG	
XF down R	ATGCGATATTCGTCACCTACAA	

### Pre-absorbed immunoproteomics method

#### Precipitation of bacterial proteins

GD201008-001 total cell protein precipitations were performed as described previously [24]. Briefly, 30 mL exponential growth cultures were centrifuged at 10, 000 × *g* for 15 min at 4°C and washed twice in PBS. Pellets were resuspended in Mutanolysin working buffer (30 mM Tris–HCl, 3 mM MgCl_2_, 25% sucrose) containing 125U/mL Mutanolysin (Sigma) and incubated for 90 minutes at 37°C. The components of solution B (7 M urea, 2 M thiourea, 4% CHAPS, and 65 mM DTT; GE Healthcare) were added directly into the mixed solution. The turbid solution gradually became transparent and was then sonicated in an ice bath for 50 cycles of 5 s on/10 s off at 100 W. After 30 min incubation at 25°C, unbroken cells was removed by centrifugation at 10,000 × g for 15 min at 4°C. Proteins in the supernatant were precipitated in 10% pre-chilled trichloroacetic acid (TCA) and incubated in ice-water for 30 min. After centrifugation at 10,000 × g for 10 min at 4°C, the pellet was resuspended in 10 mL of pre-chilled acetone and washed twice. The final pellet was dried in air.

#### Isoelectric focusing (IEF)

IEF was performed using the Ettan IPGphor-3 IEF system (GE Healthcare) with 13 cm (Immobiline DryStrip, pH 4–7; GE Healthcare) gel strips. Prior to rehydration, the precipitated proteins were treated using the 2-D Clean-up Kit (GE Healthcare) to remove contaminants that interfere with electrophoresis. IPG strips were rehydrated overnight at room temperature (RT) with rehydration solution (7 M urea, 2 M thiourea, 2% CHAPS, 0.2% DTT, 0.5% IPG buffer (pH 4–7), and 0.002% Bromophenol blue. Each strip was loaded with 200 μg of protein and IEF was carried out at 20°C for 12 h (maximum voltage of 8,000 V, maximum current of 50 μA/IPG strip, total 28,000 Vh).

#### 2D SDS-PAGE

Prior to 2D SDS-PAGE, each IPG strip was washed in equilibration buffer 1 (375 mM Tris–HCl pH 8.8, 6 M urea, 2% SDS, 2% DTT) for 15 min followed by equilibration buffer 2 (375 mM Tris–HCl pH 8.8, 6 M urea, 2% SDS, 2.5% iodoacetamide) for 15 min. Each IPG strip plus a SDS-PAGE Molecular Weight Standard (Invitrogen) was loaded on a homogeneous 12% polyacrylamide gel and sealed with 1% agarose. Electrophoresis was performed at 15°C with an initial voltage of 110 V for 30 min, followed by 220 V until the tracking dye reached the gel bottom. All gels were stained with Coomassie brilliant blue G-250 according to the manufacturer’s instructions (GE Healthcare). Each 2D IEF/SDS-PAGE experiment was repeated three times.

#### Preparation of hyperimmune sera and “pre-absorbed” sera

Rabbits were first confirmed to be negative for *S. agalactiae* antibodies using an indirect enzyme-linked immunosorbent assay (ELISA) developed in-house. Subsequently, these rabbits were immunized with formaldehyde-inactivated *S. agalactiae* GD201008-001 using Montanide ISA 206 VG (SEPPIC Co. Ltd.) as the adjuvant. Two doses of 1.0 × 10^9^cells/rabbit were administered by intramuscular injections at 3-week intervals. Sera from immunized rabbits were collected before the first and after the second immunizations. The antibody titers of the sera were evaluated by indirect ELISA.

The absorption protocol used was as described by Mittal et al. [34]. Briefly, exponential cultures of *S. agalactiae* GD201008-001 were centrifuged at 3,000 × g for 15 min at 4°C, and then washed twice in PBS. A total of 1.0 × 10^9^ bacteria were suspended in 100 μL of GD201008-001 hyperimmune serum, incubated for 2 h at 37°C, and then overnight at 4°C. Bacteria were pelleted by centrifugation at 10,000 × g for 30 min. The supernatant was collected and used as the “pre-absorbed” serum in Western blotting analyses.

#### Western blotting

The protein samples from each SDS-PAGE gel were transferred onto PVDF membranes (GE Healthcare) using a semi-dry blotting apparatus (TE77, GE Healthcare) for 2 h at 0.65 mA/cm^2^. The membrane was then blocked with 5% (w/v) skimmed milk in 50 mM Tris–HCl buffer (pH 7.4) containing 0.05% Tween 20 (TBST) for 2 h at RT. The blocked membrane was then incubated with GD201008-001 hyperimmune serum or “pre-absorbed” serum (1: 200 dilution) for 2 h at RT and washed three times with TBST (10 min per wash). The membrane was incubated with HRP-goat anti rabbit IgG (1:10,000 dilution; Boster) at RT for 1 h, washed three times with TBST, and developed by adding DAB (Tiangen) until the optimum color was obtained. Western blotting was repeated in triplicate.

#### MALDI-TOF MS and database searching

Proteins identified from the 2-DE blots as potential surface proteins were excised from duplicate SDS-PAGE gels and subjected to in-gel trypsin digestion and MALDI-TOF MS analysis (TOF Ultraflex II mass spectrometer, BrukerDaltonics). Peptide mass fingerprinting data (PMF) were analyzed using the MASCOT server (www.matrixscience.com). MASCOT searches were used to identify significant peptides and for determination of the combined peptide scores. The extent of sequence coverage, number of matched peptides, and the score probability obtained from the PMF data were all used to validate protein identification. Low-scoring proteins were either verified manually or rejected.

#### Bioinformatics analysis

Sequences of the identified proteins were searched using the BLASTX server (http://www.ncbi.nlm.nih.gov/BLASTX/) to identify homologous sequences. The PSORT server (http://www.psort.org/) program was used to predict protein subcellular localizations.

### Construction of the mutant Δxf

Two DNA fragments flanking the *xf* gene were amplified by PCR from the genome of GD201008-001 with the primers XF A/B and primers XF C/D with incorporated *Sal*I and *Sma*I restriction enzyme sites, respectively in primers A and D (Table 1). The resulting PCR products were mixed in equal amounts and subjected to a crossover PCR to generate a single PCR product. The crossover PCR product and the temperature-sensitive streptococcus-*E. coli* shuttle vector pSET4s were digested with appropriate enzymes, ligated and used to transform *E. coli* DH5α. The resulting pSET4S-XF plasmid was introduced into competent *S. agalactiae* GD201008-001 cells by electroporation at 2.35 KV, 200Ω and 25 μF.

The procedure for the selection of mutants generated by allelic exchange via double-crossover was performed as described previously [18] with some modifications. In the presence of the appropriate antibiotics, the temperature-sensitive plasmid can be propagated in *E. coli* at 37°C, while its replication is blocked in *Streptococcus* above 37°C. After electroporation, *S. agalactiae* containing the pSET4S-*xf* plasmid were first grown at 28°C in THY (THB supplemented with 2% yeast extract) for 4 h to recover, and then cultured in THY containing 100 μg/mL spectinomycin to mid-logarithmic growth phase. The pSET4S-*xf* plasmid in *S. agalactiae* duplicated well in the appropriate antibiotics at 28°C. The cells were then diluted and plated on TSB containing 100 μg/mL spectinomycin and cultured at 37°C. When the temperature increased from 28°C to 37°C, the replication of this vector in *S. agalactiae* is blocked immediately and the bacteria without the pSET4S-*xf* plasmid are killed by spectinomycin. Thus, at the non-permissive temperature (37°C) under antibiotic pressure, most of bacteria were destroyed and only a few survived by integrating the pSET4S-*xf* plasmid genes into their own chromosome by allelic exchange via single crossover recombination. The single bacterial colony on the 100 μg/mL spectinomycin TSB plate cultured at 37°C was transferred to 28°C in TSB liquid without antibiotics. These conditions forced loss of the spectinomycin-resistance gene in their chromosome by allelic exchange. During the complete procedure, the chromosomal *xf* gene of *S. aglactiae* was replaced by a total double-crossover event (Figure 3). The identity of the correct Δ*xf* mutants was confirmed by PCR using XF A/D, XF1/2 primers and DNA sequencing of the PCR product.

### Functional complementation of Δxf

The *xf* gene, including its own promoter, was amplified from the chromosomal DNA of GD201008-001 by PCR using the primers C XF F and C XF R (Table 1). The PCR product was cut with *BamH*I/*Pst*I and ligated into the *BamH*I/*Pst*I digested pSET2 [19] to generate the recombinant plasmid pSET2-CΔ*xf*. The identity of the correct pSET2-CΔ*xf* vector was confirmed by sequencing using the primers C XF F and C XF R. The pSET2-CΔ*xf* plasmid was then used to electrotransform the Δ*xf* mutant to create the complemented strain of ΔXF (designated CΔ*xf*) on THB agar under spectinomycin selection pressure.

### Quantitative real-time reverse transcription PCR (qRT-PCR)

*S. agalactiae* strains GD201008-001, Δ*xf, C*Δ*xf* were cultured overnight in TSB at 37°C and isolated RNA with an E.Z.N.A.™ Bacterial RNA isolation kit (OMEGA, Beijing, China). The cDNA synthesis was performed using the PrimeScript™ RT reagent kit with DNA eraser (TaKaRa, Dalian, China) according to the manufacturer’s instructions. The mRNA levels were measured using two-step relative qRT-PCR. The glyceraldehyde-3-phosphate dehydrogenase (GAPDH) housekeeping gene was amplified as an internal control. The specific primers used for the various RT-PCR assays are listed in Table 1. The SYBR Green PCR method was performed using the AceQ™ qPCR SYBR™ Green Master Mix (Vazyme Biotech Co., Ltd.). Reactions were carried out in triplicate and repeated three times. An ABI 7300 RT-PCR system (Applied Biosystems) was used for relative qRT-PCR. Dissociation analysis of amplification products was performed at the end of each PCR to confirm that only one PCR product was amplified and detected. The comparative cycle threshold method (2^−ΔΔCT^method) [35] was used to analyze the mRNA levels.

### Growth characteristics of mutant strains

The wild-type strain GD201008-001, mutant strain Δ*xf* and the complementation strain CΔ*xf* were separately inoculated into flasks containing 100 mL TSB media, and incubated at 37°C and 28°C. Samples of culture were monitored at 1 h intervals for 13 h using a spectrophotometer (Bio-Rad, USA) at an absorbance of 600 nm. The un-inoculated TSB served as the blank control. The experiments were repeated twice.

### Cell adherence assay

The cell adherence and invasion assay was performed as previously described [36]. Briefly, the human laryngeal cell line HEp-2 cells was cultured in RPMI 1640 media (Invitrogen, USA) in 24-well tissue culture plates, supplemented with 10% fetal calf serum (FCS), and maintained at 37°C in a 5% CO_2_ humidified incubator. The wild-type strain GD201008-001, mutant strain Δ*xf* and complementation strain CΔ*xf* were cultured separately in TSB medium to the logarithmic phase and diluted to the appropriate density to give a MOI of 10 in RPMI 1640 medium (without FCS). The monolayers were washed by RPMI 1640 medium (without FCS) and incubated with diluted bacteria. The plates were centrifuged at 800 × g for 10 min at RT before incubation for 2 h at 37°C. After incubation, the medium was removed from each well and monolayers were washed five times using PBS (pH 7.4). For the cellular invasion assay, the extracellular bacteria were eliminated by additional incubation of the monolayers with experimental medium containing gentamicin (100 μg/mL) for 120 min at 37°C and the plate was washed five times using PBS. HEp-2 cells were detached from plates by treatment with 100 μL of 0.25% trypsin–0.1% EDTA prepared in PBS (pH 7.4). The cells were lysed by the addition of 900 μL ddH_2_O and appropriate dilutions were plated on TSB agar plates for enumeration of the bacteria adhering or invading to HEp-2 cells. Negative control wells containing only cells were used in all experiments. All assays were performed in triplicate and repeated at least three times.

### Animal experiments

The feeding and care of zebrafish supplied by the Pearl River Fishery Research Institute of the Chinese Academy of Fishery Science were performed as described by Neely et al. [31]. The virulence of streptococcus was evaluated in a zebrafish model as described previously [32]. Specifically, 50% lethal doses (LD_50_) were ascertained to determine the differences in virulence between the wild-type and mutant strains [33]. Prior to inoculation of fish, cultures (*S. agalactiae* strains GD201008-001, Δ*xf* and *C*Δ*xf*) were collected, cultured at 37°C to the logarithmic growth phase, washed twice in TSB and adjusted to the appropriate doses (CFU/fish). Zebrafish were anesthetized with tricaine methanesulfonate (MS-222) (Hangzhou Animal Medicine Factory) at a concentration of 80 mg/L. The bacterial dose (CFU) contained in the injected inoculum was confirmed at the time of infection by plating onto TSB agar. Control fish were injected with TSB. Fifteen fish were used to test each dose. Mortality was monitored until 7 days post-infection. The experiment was repeated three times and the results were used to calculate the LD_50_ values using the method of Reed and Muench [37]. The experimental animal use license for the zebrafish studies was approved by the Science and Technology Agency of Jiangsu Province (China).

### Statistical analyses

All the statistical analysis was performed using SPSS version 17.0 (SPSS Inc., Chicago, IL). Differences between the mean values among groups was first evaluated by one-way analysis of variance (ANOVA) and then by pairwise comparison of the mean values between the two groups, followed by Tukey’s student rank test. Differences with a *P*-value of <0.05 were considered significant, and a *P*-value of <0.001 was considered highly significant.

## Additional file

Additional file 1: Table S1. The MALDI-TOF MS and database screening results of XF spot.

## Abbreviations

PAIM: Pre-absorbed immunoproteomic method
Srr: Serine-rich repeat protein
GBS: Group B Streptococcus
MALDI-TOF MS: Matrix-assisted laser desorption ionization-time of flight mass spectrometry
GAPDH: Glyceraldehyde 3-phosphate dehydrogenase
HEp-2 cell: Human epidermoid cancer cells
MOI: Multiplicity of infection
TSB: Tryptic soy broth
DTT: Dichlorodiphenyltrichloroethane
CHAPS: 3-[(3- cholamidopropyl) dimethylammonio]-1-propanesulfonate
TCA: Trichloroacetic acid
LB: Lysogeny broth
IEF: Isoelectric focusing
SDS-PAGE: Sodium dodecyl sulfate polyacrylamide gel electrophoresis
LD_50_: 50% lethal doses
ELISA: Enzyme-linked immunosorbent assay
THB: Todd-Hewitt Broth
RPMI 1640 medium: Roswell Park Memorial Institute1640 medium
CFU: Colony-Forming Units
