# Supplementary material for: Identification of a virulence-related surface protein XF in piscine Streptococcus agalactiae by pre-absorbed immunoproteomics
Source: BMC Vet Res. 2014 Oct 26;10:259. doi: 10.1186/s12917-014-0259-7 (PMC4219122; doi:10.1186/s12917-014-0259-7)
Supplement: Additional file 1: Table S1. — The MALDI-TOF MS and database screening results of XF spot. [file 12917_2014_259_MOESM1_ESM.pdf]

Analysis Information

**Report Type**	Protein-Peptide Summary by Spot	**Analysis Type**	MALDI-TOF MS
**Sample Set Name**	Project sample_20120802	**Database**	NCBInr
**Analysis Name**	R12007-6sb	**Creation Date**	08/07/2012 17:11:45
**Reported By**	08/08/2012 10:52:30 - admin	**Last Modified**	08/07/2012 18:45:37

MS Acq. : Proc. Methods (Unspecified) : (Unspecified)

Interpretation Method (Unspecified)

**Gel Idx/Pos**	111/E10	**Instr./Gel Origin**	ak044/Sample Project 20120802	**Process Status**	Analysis Succee
**Plate [#] Name**	[1] Sample Project 20120802	**Instrument Sample Name**		**Spectra**	11

Rank	Accession No.	Protein Name	Protein PI	Protein MW	Protein Score	Protein Score C. I. %	otal Ion Score	Total Ion C. I. %	Pep. Count
1	gi|77411051	cell wall surface anchor family protein [Streptococcus agalactiae CJB111]	5.28	113612.1	527	100	493	100	11

Peptide Information

Calc. Mass	Obsrv. Mass	± da	± ppm	Start Seq.	End Seq.	Sequence	Ion Score	C. I. %	Modification	Rank
1010.5417	1010.579	0.0373	37	431	438	LPVWPDQR				
1010.5417	1010.579	0.0373	37	431	438	LPVWPDQR	49	77.711		
1692.8551	1692.9396	0.0845	50	450	464	FTATLNNQVASIEER				
1692.8551	1692.9396	0.0845	50	450	464	FTATLNNQVASIEER	103	100		
1702.8395	1702.8732	0.0337	20	465	479	VQYNSPSVTEHTNVK				
2056.8772	2056.9622	0.085	41	621	638	YFYGYTQVDGESDANAE K				
2184.9722	2185.0713	0.0991	45	621	639	YFYGYTQVDGESDANAE KK				
2184.9722	2185.0713	0.0991	45	621	639	YFYGYTQVDGESDANAE KK	61	98.534		
2222.0823	2222.1792	0.0969	44	489	507	IDDEKQTETYITQINPEGK				
2222.0823	2222.1792	0.0969	44	489	507	IDDEKQTETYITQINPEGK	74	99.936		
2692.3142	2692.4321	0.1179	44	349	372	SENLVLNFDFSIASVNEG DFFTVK				
3335.7158	3335.9402	0.2244	67	388	417	VQDIIDTSGQLLATGSYS PLTHNITYIWTK				
3335.7158	3335.9402	0.2244	67	388	417	VQDIIDTSGQLLATGSYS PLTHNITYIWTK	82	99.989		

3417.6882	3417.9163	0.2281	67	508	540	EMYFASGLGNLYTIIGSD GTSGSPVNLLNAEVK	36	0		
3417.7241	3417.9163	0.1922	56	255	285	MSNESLITPEKPQVMIAS			Oxidation (M)[1]	

						DKTGNETLTPTIR				
3454.4023	3454.6074	0.2051	59	548	577	NLTDSMDQNYDSTEFEDVTSQYSYTNDGSK				
3454.4023	3454.6074	0.2051	59	548	577	NLTDSMDQNYDSTEFEDVTSQYSYTNDGSK	89	99.998		

2	gi|76787518	cell wall surface anchor family protein [Streptococcus agalactiae A909]	5.35	124907.9	521	100	493	100	11

Peptide Information

Calc. Mass	Obsrv. Mass	± da	± ppm	Start Seq.	End Seq.	Sequence	Ion Score	C. I. %	Modification	Rank
1010.5417	1010.579	0.0373	37	431	438	LPVWPDQR				
1010.5417	1010.579	0.0373	37	431	438	LPVWPDQR	49	77.711		
1692.8551	1692.9396	0.0845	50	450	464	FTATLNNQVASIEER				
1692.8551	1692.9396	0.0845	50	450	464	FTATLNNQVASIEER	103	100		
1702.8395	1702.8732	0.0337	20	465	479	VQYNSPSVTEHTNVK				
2056.8772	2056.9622	0.085	41	621	638	YFYGYTQVDGESDANAEK				
2184.9722	2185.0713	0.0991	45	621	639	YFYGYTQVDGESDANAEKK				
2184.9722	2185.0713	0.0991	45	621	639	YFYGYTQVDGESDANAEKK	61	98.534		
2222.0823	2222.1792	0.0969	44	489	507	IDDEKQTETYITQINPEGK				
2222.0823	2222.1792	0.0969	44	489	507	IDDEKQTETYITQINPEGK	74	99.936		
2692.3142	2692.4321	0.1179	44	349	372	SENLVLNFDFSIASVNEGDFFTVK				
3335.7158	3335.9402	0.2244	67	388	417	VQDIIDTSGQLLATGSYSPLTHNITYIWTK				
3335.7158	3335.9402	0.2244	67	388	417	VQDIIDTSGQLLATGSYSPLTHNITYIWTK	82	99.989		
3417.6882	3417.9163	0.2281	67	508	540	EMYFASGLGNLYTIIGSDGTSGSPVNLLNAEVK	36	0		
3417.7241	3417.9163	0.1922	56	255	285	MSNESLITPEKPQVMIASDKTGNETLTPTIR			Oxidation (M)[1]	
3454.4023	3454.6074	0.2051	59	548	577	NLTDSMDQNYDSTEFEDVTSQYSYTNDGSK				
3454.4023	3454.6074	0.2051	59	548	577	NLTDSMDQNYDSTEFEDVTSQYSYTNDGSK	89	99.998		

3	gi|341576044	cell wall surface anchor family protein [Streptococcus agalactiae FSL S3-026]	4.99	72599.2	480	100	432	100	9

Peptide Information

Calc. Mass	Obsrv. Mass	± da	± ppm	Start Seq.	End Seq.	Sequence	Ion Score	C. I. %	Modification	Rank

1010.5417	1010.579	0.0373	37	435	442	LPVWPDQR				

Project\Project sample_2012 1010.579

1010.5417	1010.579	0.0373	37	435	442	LPVWPDQR	49	77.711		
1692.8551	1692.9396	0.0845	50	454	468	FTATLNNQVASIEER				
1692.8551	1692.9396	0.0845	50	454	468	FTATLNNQVASIEER	103	100		
1702.8395	1702.8732	0.0337	20	469	483	VQYNSPSVTEHTNVK				
2222.0823	2222.1792	0.0969	44	493	511	IDDEKQTETYITQINPEGK				
2222.0823	2222.1792	0.0969	44	493	511	IDDEKQTETYITQINPEGK	74	99.936		
2692.3142	2692.4321	0.1179	44	353	376	SENLVLNFDFSIASVNEG DFFTVK				
3335.7158	3335.9402	0.2244	67	392	421	VQDIIDTSGQLLATGSYS PLTHNITYIWTK				
3335.7158	3335.9402	0.2244	67	392	421	VQDIIDTSGQLLATGSYS PLTHNITYIWTK	82	99.989		
3417.6882	3417.9163	0.2281	67	512	544	EMYFASGLGNLYTIIGSD GTSGSPVNLLNAEVK	36	0		
3417.7241	3417.9163	0.1922	56	259	289	MSNESLITPEKPQVMIAS DKTGNETLTPTIR			Oxidation (M)[1]	
3454.4023	3454.6074	0.2051	59	552	581	NLTDSMDQNYDSTEFED VTSQYSYTNDGSK				
3454.4023	3454.6074	0.2051	59	552	581	NLTDSMDQNYDSTEFED VTSQYSYTNDGSK	89	99.998		

4	gi|339301130	serine-rich glycoprotein adhesin, partial [Streptococcus agalactiae ATCC 13813]	5.19	91119.5	461	100	432	100	8

Peptide Information

Calc. Mass	Obsrv. Mass	± da	± ppm	Start Seq.	End Seq.	Sequence	Ion Score	C. I. %	Modification	Rank
1010.5417	1010.579	0.0373	37	431	438	LPVWPDQR				
1010.5417	1010.579	0.0373	37	431	438	LPVWPDQR	49	77.711		
1692.8551	1692.9396	0.0845	50	450	464	FTATLNNQVASIEER				
1692.8551	1692.9396	0.0845	50	450	464	FTATLNNQVASIEER	103	100		
1702.8395	1702.8732	0.0337	20	465	479	VQYNSPSVTEHTNVK				
2222.0823	2222.1792	0.0969	44	489	507	IDDEKQTETYITQINPEGK				
2222.0823	2222.1792	0.0969	44	489	507	IDDEKQTETYITQINPEGK	74	99.936		
3335.7158	3335.9402	0.2244	67	388	417	VQDIIDTSGQLLATGSYS PLTHNITYIWTK				
3335.7158	3335.9402	0.2244	67	388	417	VQDIIDTSGQLLATGSYS PLTHNITYIWTK	82	99.989		
3417.6882	3417.9163	0.2281	67	508	540	EMYFASGLGNLYTIIGSD GTSGSPVNLLNAEVK	36	0		
3417.7241	3417.9163	0.1922	56	255	285	MSNESLITPEKPQVMIAS DKTGNETLTPTIR			Oxidation (M)[1]	

Calc. Mass	Obsrv. Mass	± da	± ppm	Start Seq.	End Seq.	Sequence	Ion Score	C. I. %	Modification	Rank
3454.4023	3454.6074	0.2051	59	548	577	NLTDSMDQNYDSTEFED VTSQYSYTNDGSK				
3454.4023	3454.6074	0.2051	59	548	577	NLTDSMDQNYDSTEFED VTSQYSYTNDGSK	89	99.998		

5	gi|315639131	DNA repair protein RadA [Campylobacter upsaliensis JV21]	6.04	49589.1	63	0	10

Peptide Information

Calc. Mass	Obsrv. Mass	± da	± ppm	Start Seq.	End Seq.	Sequence	Ion Score	C. I. %	Modification	Rank
871.5247	871.5613	0.0366	42	38	45	AEQVAILK				
995.4978	995.5739	0.0761	76	410	417	EAKMQNFK				
1010.5226	1010.579	0.0564	56	194	201	ELTFELMK				
1010.5226	1010.579	0.0564	56	194	201	ELTFELMK				
1025.5011	1025.5945	0.0934	91	321	329	SATGYEKNR				
1026.5177	1026.5642	0.0465	45	194	201	ELTFELMK			Oxidation (M)[7]	
2057.1067	2056.9622	-0.1445	-70	341	359	LEIPLGHYDVFINVSGGV K				
2185.2017	2185.0713	-0.1304	-60	340	359	KLEIPLGHYDVFINVSGG VK				
2185.2017	2185.0713	-0.1304	-60	340	359	KLEIPLGHYDVFINVSGG VK				
2228.1267	2228.312	0.1853	83	256	276	FGNTSEVGIFEMSPKGLI SAK			Oxidation (M)[12]	
2256.0205	2256.1667	0.1462	65	1	19	MAKNKPIFECEACGNQQ SK			Carbamidomethyl (C)[10,13], Oxidation (M)[1]	
3317.7263	3317.9065	0.1802	54	164	193	QDYEFLIIDSIQTLYSSKIA SAAGSITQVR				
3354.6919	3354.9377	0.2458	73	288	319	SSAGSALSVVMEGSRAL VLEIQALVCESSYPK			Carbamidomethyl (C)[26], Oxidation (M)[11]	

6	gi|161519927	MlrC domain-containing protein [Burkholderia multivorans ATCC 17616]	5.55	54478.6	62	0	11

Peptide Information

Calc. Mass	Obsrv. Mass	± da	± ppm	Start Seq.	End Seq.	Sequence	Ion Score	C. I. %	Modification	Rank
878.473	878.5519	0.0789	90	174	181	ADALVAYR				
1010.4976	1010.579	0.0814	81	505	513	TKPMGDAFK			Oxidation (M)[4]	
1010.4976	1010.579	0.0814	81	505	513	TKPMGDAFK			Oxidation (M)[4]	
1032.5031	1032.5403	0.0372	36	448	457	VGGVEPEAMK			Oxidation (M)[9]	
1562.7996	1562.8235	0.0239	15	444	457	NLYRVGGVEPEAMK				
1691.8357	1691.8378	0.0021	1	499	513	LAHGMRTKPMGDAFK			Oxidation (M)[5,10]	

Calc. Mass	Obsrv. Mass	± da	± ppm	Start Seq.	End Seq.	Sequence	Ion Score	C. I. %	Modification	Rank
1715.0074	1714.8883	-0.1191	-69	195	211	AAALLERLLAGGGPLHR				
1880.99	1881.0172	0.0272	14	6	22	TSRTPTVLPGFSLMTTR			Oxidation (M)[14]	
1901.8964	1902.0853	0.1889	99	40	56	AAYANFERGEGFPAMVR			Oxidation (M)[15]	
2057.0923	2056.9622	-0.1301	-63	216	233	LPFLIPINGMCTLLDPAR			Carbamidomethyl (C)[11], Oxidation (M)[10]	
3417.7988	3417.9163	0.1175	34	9	39	TPTVLPGFSLMTTRILIAG				

Project\Project sample_2012 3417.9163

Calc. Mass	Obsrv. Mass	± da	± ppm	Start Seq.	End Seq.	Sequence	Ion Score	C. I. %	Modification	Rank
						FQHETNTFAPTR				
3417.7988	3417.9163	0.1175	34	9	39	TPTVLPGFSLMTTRILIAG FQHETNTFAPTR				
3489.5981	3489.5234	-0.0747	-21	394	425	FEVVAVSDGVCRYDGPM MNGMLADVGPVACLK			Carbamidomethyl (C)[11,30], Oxidation (M)[17,18]	

7	gi|322418284	NAD-dependent epimerase/dehydratase [Geobacter sp. M18]	5.93	37907.1	62	0	9	

Peptide Information

Calc. Mass	Obsrv. Mass	± da	± ppm	Start Seq.	End Seq.	Sequence	Ion Score	C. I. %	Modification	Rank
1032.5109	1032.5403	0.0294	28	49	57	QLEGEPGFR				
1436.7169	1436.7699	0.053	37	49	60	QLEGEPGFRFEK				
1628.8755	1628.9344	0.0589	36	259	272	IYNIGNNNPVELLR				
1691.9116	1691.8378	-0.0738	-44	200	214	AILEGRPIDVFNYGK				
1702.9374	1702.8732	-0.0642	-38	273	287	FIEVLEQALGKEAQK				
1702.9374	1703.036	0.0986	58	273	287	FIEVLEQALGKEAQK				
2185.0596	2185.0713	0.0117	5	218	235	DFTYIDDIVEGVCRVIDR			Carbamidomethyl (C)[13]	
2185.0596	2185.0713	0.0117	5	218	235	DFTYIDDIVEGVCRVIDR			Carbamidomethyl (C)[13]	
2409.2102	2409.3643	0.1541	64	180	199	FFTVYGPWGRPDMALFL FTK			Oxidation (M)[13]	
2498.2576	2498.4287	0.1711	68	310	331	DVGFRPATSIEDGVGRFV AWYR				
3454.667	3454.6074	-0.0596	-17	92	121	YSLINPYAYVDSNLSGFM NILEGCRHHGVK			Carbamidomethyl (C)[24]	
3454.667	3454.6074	-0.0596	-17	92	121	YSLINPYAYVDSNLSGFM NILEGCRHHGVK			Carbamidomethyl (C)[24]	

8	gi|300870460	unnamed protein product [Brachyspira pilosicoli 95/1000]	10	7701.5	59	0	3	

Peptide Information

Calc. Mass	Obsrv. Mass	± da	± ppm	Start Seq.	End Seq.	Sequence	Ion Score	C. I. %	Modification	Rank
1010.6357	1010.579	-0.0567	-56	32	40	LPGDIVIKR				
1010.6357	1010.579	-0.0567	-56	32	40	LPGDIVIKR	28	0		
3334.8201	3335.0259	0.2058	62	41	69	ENFTFAFPIVTSIIASIVLS FIMWIISKF				

Calc. Mass	Obsrv. Mass	± da	± ppm	Start Seq.	End Seq.	Sequence	Ion Score	C. I. %	Modification	Rank
3359.8477	3359.9097	0.062	18	40	68	RENFTFAFPIVTSIIASIVL SFIMWIISK			Oxidation (M)[24]	
3359.8477	3359.9097	0.062	18	40	68	RENFTFAFPIVTSIIASIVL SFIMWIISK			Oxidation (M)[24]	

9	gi|317474038	N-acetylneuraminate synthase [Bacteroides eggerthii 1_2_48FAA]	5.91	37134	59	0	8

Peptide Information

Calc. Mass	Obsrv. Mass	± da	± ppm	Start Seq.	End Seq.	Sequence	Ion Score	C. I. %	Modification	Rank
1562.806	1562.8235	0.0175	11	23	37	QLIDAAAEAGVDYVK				
1628.8901	1628.9344	0.0443	27	250	264	AMVSAIRNIEQAIGR				
1858.007	1858.0288	0.0218	12	233	249	NLPGPDHKASLEPLELK				
2057.1501	2056.9622	-0.1879	-91	3	22	HTLIIAEAGVNHNGSLVLA K				
2185.2451	2185.0713	-0.1738	-80	2	22	KHTLIIAEAGVNHNGSLVL AK				
2185.2451	2185.0713	-0.1738	-80	2	22	KHTLIIAEAGVNHNGSLVL AK				
3317.6294	3317.9065	0.2771	84	293	322	GETFTENNLAVKRPGTGI SPMCWPEVIGEK			Carbamidomethyl (C)[22]	
3454.5569	3454.6074	0.0505	15	166	194	KEQITVLHCNTEYPTPMC DVNLSAMSEMR			Carbamidomethyl (C)[9,18]	
3454.5569	3454.6074	0.0505	15	166	194	KEQITVLHCNTEYPTPMC DVNLSAMSEMR			Carbamidomethyl (C)[9,18]	
3489.7493	3489.5234	-0.2259	-65	133	165	IASFHEPVILSTGMSTLEE IESAMGVLTDNGLK				

10	gi|46203607	COG0330: Membrane protease subunits, stomatin/prohibitin homologs [Magnetospirillum magnetotacticum MS-1]	8.01	15264.9	58	0	6

Peptide Information

Calc. Mass	Obsrv. Mass	± da	± ppm	Start Seq.	End Seq.	Sequence	Ion Score	C. I. %	Modification	Rank
1010.5226	1010.579	0.0564	56	92	99	IFLETMEK				
1010.5226	1010.579	0.0564	56	92	99	IFLETMEK				
1026.5177	1026.5642	0.0465	45	92	99	IFLETMEK			Oxidation (M)[6]	
1040.4644	1040.5322	0.0678	65	32	40	NEAETYASR				
1691.8347	1691.8378	0.0031	2	32	46	NEAETYASRVVPEAR				
2409.2158	2409.3643	0.1485	62	52	75	ALQGAEAYVAQATADAT GQAARFK				
3335.7495	3335.9402	0.1907	57	2	31	VQLTSVNPPPEVRPAFID				

						VNAAQQYAQQVR
3335.7495	3335.9402	0.1907	57	2	31	VQLTSVNPPPEVRPAFID
						VNAAQQYAQQVR
3455.9221	3455.7153	-0.2068	-60	100	134	VLGSVHKVIIDQSGGVSG
						ANVAGVLPVLPLTESSR
